# Cause of death and the autopsy rate in an elderly population

**DOI:** 10.1007/s00428-023-03571-0

**Published:** 2023-06-03

**Authors:** Bartholomeus G. H. Latten, Bela Kubat, Piet A. van den Brandt, Axel zur Hausen, Leo J. Schouten

**Affiliations:** 1 Department of Pathology, Maastricht University Medical Centre+, P. Debyelaan 25, 6229 HX Maastricht, The Netherlands; 2 Department of Epidemiology, GROW–School for Oncology and Reproduction, Maastricht University, P.O. Box 616, 6200 MD Maastricht, The Netherlands; 3 Department of Pathology, GROW–School for Oncology and Reproduction, Maastricht University, P. Debyelaan 25, 6229 HX Maastricht, The Netherlands

**Keywords:** Autopsy, Quality of healthcare, Autopsy rate, Death certificate, Cause of death

## Abstract

Autopsy rates are declining, while major discrepancies between autopsies and clinical diagnoses remain. Still, little is known about the impact of suspected underlying diseases, for example, a diagnosis of cancer, on the autopsy rate. The aim of this study was to investigate the relation between the clinical cause of death, a history of cancer, and the medical autopsy rate using data from the Netherlands Cohort Study on Diet and Cancer (NLCS), a large prospective cohort study with a long follow-up. The NLCS is a prospective study initiated in 1986 and includes 120,852 persons (58,279 males and 62,573 females), 55–69 years of age at the time of enrollment. The NLCS was linked with the Dutch Nationwide Pathology Databank (PALGA), the Dutch Population Register (GBA), the Netherlands Cancer Registry, and the causes of death registry (Statistics Netherlands). If applicable, the 95% confidence intervals were calculated. During the follow-up of the NLCS, 59,760 deaths were recorded by linkage with the GBA from 1991 until 2009. Of these, a medical autopsy was performed on 3736 deceased according to linkage with PALGA, resulting in an overall autopsy rate of 6.3%. Major variations in the autopsy rate were observed according to the cause of death. The autopsy rate increased according to the number of contributing causes of death. Lastly, a diagnosis of cancer affected the autopsy rate. The clinical cause of death and a history of cancer both influenced the medical autopsy rate in a large national cohort. The insight this study provides may help clinicians and pathologists counteracting the further downfall of the medical autopsy.

## Introduction

Medical autopsy stood at the basis of many important advances in medicine and health care [1, 2]. Nonetheless, over the last decades, a steady worldwide decline of autopsies has been observed [3-6]. Several explanations for this decline have been proposed, such as the attitude of the public, clinicians, and pathologists [7-9], lack of education in medical curricula [10], a shift in care for older or sicker people from the hospital towards long-term facilities [11], the financial aspects [12], and advances in minimally invasive alternatives [13-17]. A recent Dutch study suggests that a key aspect for not requesting an autopsy is the assumption that the cause of death is already known [18]. However, major discrepancies between autopsies and clinical diagnoses remain, even in this modern era [19-25]. Of interest, throughout the year 2020, autopsies have seen a small revival due to COVID-19 [26, 27]. Still, little is known about the impact of clinically suspected underlying diseases, e.g., a cancer diagnosis, on the autopsy rate.

The aim of this study was to investigate the relation of the clinical cause of death and a history of cancer to the autopsy rate, using data from the Netherlands Cohort Study on Diet and Cancer (NLCS). The NLCS is a large prospective cohort study with a long follow-up that has been linked to multiple population and health registries, among them nationwide data from Statistics Netherlands (CBS) and the Dutch Nationwide Pathology Databank (PALGA) [28].

## Method

The NLCS was initiated in 1986 and has been described in detail elsewhere [29]. In brief, this prospective study encompasses 120,852 persons (58,279 males and 62,573 females), 55–69 years of age at the time of enrollment, living in 204 municipalities located throughout the Netherlands. Participants of the study consented by completing and returning the questionnaire.

The vital status was obtained by record linkage with the Central Bureau of Genealogy (CBG) and the automated municipal population registries (GBA), which record information of all inhabitants in the Netherlands, including death, birth, and migration. Information regarding vital status was available for 99.7% of the cohort on the 31st of December 2014.

The deceased participants of the NLCS were followed up for the performance of a medical autopsy by record linkage with the PALGA database using a linkage protocol described by van den Brandt et al. [30]. In the PALGA, database autopsies are coded and are therefore clearly distinguishable from other pathology reports. Although the autopsy conclusions were available, these were not used for this study. The autopsy rate was calculated as the number of autopsies divided by the number of deaths.

The cause of death (COD) per individual was established by record linkage with the cause of death registry maintained by Statistics Netherlands. For this analysis, data was used for the period 1991–2009 because PALGA had nationwide coverage since 1991 [28], and the linkage to the cause of death registry was completed until the 31st of December 2009 at the moment of analysis. Statistics Netherlands is able to request an autopsy report to adjust the COD as stated on the death certificate; however, this is only done in a limited number of cases. Therefore, we can assume that the data consists mostly of the suspected COD as determined by the physician, with little to no adjustments due to the results of the postmortem, i.e., the clinical COD.

The underlying COD was grouped according to the BELDO list [31]. The BELDO list is a shortlist that aims to provide a quick overview of the most relevant (i.e., common) COD in the Netherlands [32]. The list was created by the CBS in 1993, was based on the International Classification of Diseases (ICD), and has been the basis for the European Shortlist, as created by Eurostat in August 1998. For an overview of the ICD-9 and ICD-10 codes of the BELDO list, see Table 2. The two groups of COD with the highest and the lowest autopsy rates were further investigated by examining the individual COD within these groups.

**Table 1:** Demographics, number of deaths and autopsies in the Netherlands Cohort Study (1991–2009)

	Males		Females		Total	
Follow-up (1991–2009)	*N*	%	*N*	%	*N*	%
Died	33058	61.3%	26702	44.1%	59760	52.2%
Emigrated	172	0.3%	108	0.2%	280	0.2%
Lost-to follow-up	1	0.0%	0	0.0%	1	0.0%
Alive at 31–12-2009	20667	38.3%	33693	55.7%	54360	47.5%
Total	53898	100.0%	60503	100.0%	114401	100.0%
Age at baseline in 1991						
55–59 years	2670	5.0%	3054	5.0%	5724	5.0%
60–64 years	20608	38.2%	22296	36.9%	42904	37.5%
65–69 years	18685	34.7%	20980	34.7%	39665	34.7%
70–74 years	11935	22.1%	14173	23.4%	26108	22.8%
Total	53898	100.0%	60503	100.0%	114401	100.0%
Age at death 1991–2009						
55–64 years	885	2.7%	497	1.9%	1382	2.3%
65–74 years	10361	31.3%	5808	21.8%	16169	27.1%
75–84 years	17698	53.5%	14579	54.6%	32277	54.0%
85–94 years	4114	12.4%	5818	21.8%	9932	16.6%
Total	33,058	100.0%	26702	100.0%	59760	100.0%
Autopsy rate 1991–2009 by age (*N* autopsies/*N* deaths)						
55–64 years	119/885	13.4%	42/497	8.5%	161/1382	11.6%
65–74 years	1071/10361	10.3%	556/5808	9.6%	1627/16169	10.1%
75–84 years	999/17698	5.6%	711/14579	4.9%	1710/32277	5.3%
85–94 years	119/4114	2.9%	119/5818	2.0%	238/9932	2.4%
Total	2308/33058	7.0%	1428/26702	5.3%	3736/59760	6.3%

Lastly, an occurrence of cancer until the 31st of December 2009 was identified by linkage with the Netherlands Cancer Registry and PALGA. The most recent record of an invasive cancer, non-melanoma skin cancer excluded, was selected and the time between the date of cancer incidence and date of death was calculated. Categories were created as follows: 0–1 days; 2–31 days; 32–183 days; 184–365 days; 1– < 2 years; 2– < 3 years; 3– < 4 years; 4– < 5 years; 5– < 10 years; 10– < 15 years; 15– < 20 years, and 20 + years. As age has an important influence on the autopsy [6], the expected number of autopsies in the group with a diagnosis of cancer was calculated using the autopsy rate per 5-year age category in the group without a diagnosis of cancer. In other words, the expected number of autopsies was corrected for age and sex. Next, the observed/expected (O/E) ratio was calculated with 95% confidence intervals.

The autopsy rate was also calculated for each individual COD according to the BELDO list, the time of death in relation to a diagnosis of cancer, and the number of different COD per case. Next, an independent sample *t*-test was conducted to compare the number of COD in deceased with and without an autopsy.

## Results

At the time of inclusion in this study (January 1st 1991), 114,401 of the 120,852 NLCS participants were still alive. The demographics, number of deaths, and autopsies in the NLCS are shown in Table 1. During the follow-up of the NLCS, 59,760 deaths were recorded by linkage with CBG and GBA from 1991 until 2009. Of these, an autopsy was performed on 3736 deceased according to linkage with PALGA, resulting in an overall autopsy rate of 6.3%. The autopsy rate varies according to age at death and sex.

The number and percentage of performed autopsies per underlying COD, as classified by the BELDO list, are shown in Table 2. The autopsy rate varies considerably among the grouped CODs. Of 181 (0,3%) included participants, the cause of death was not known, and the CBS was unable to apply a code. These cases mostly included a death abroad or administrative errors. In 16 (8.8%) of these, an autopsy was performed. In 1764 (3%), the cause of death was unclear, hence it was specified as “symptoms, signs, abnormal findings, ill-defined causes.” In 89 (5%) of these, an autopsy was performed. To our knowledge, the CBS only occasionally contacts clinicians in order to request the autopsy information for the purpose of a correct cause of death.

**Table 2:** Autopsy rate per main category of underlying cause of death (1991–2009)

Cause of death*	*N* deaths	N autopsies	Autopsy rate	ICD-10	ICD-9
Infectious and parasitic diseases	718	103	14.3%	A00–B99	040–042
Neoplasms	18688	1025	5.5%	C00–D48	140–239
Diseases of the blood(-forming organs), immunologic disorders	199	15	7.5%	D50–D89	279–289 excl. 279.8
Endocrine, nutritional and metabolic diseases	1612	74	4.6%	E00–E90	240–278
Mental and behavioral disorders	2354	19	0.8%	F00–F99	290–278
Diseases of the nervous system and the sense organs	1708	68	4.0%	G00–H95	320–389
Diseases of the circulatory system	21723	1400	6.4%	I00–I99	390–459
Diseases of the respiratory system	5701	349	6.1%	J00–J99	460–519
Diseases of the digestive system	2082	351	16.9%	K00–K93	520–579
Diseases of the skin and subcutaneous tissue	127	7	5.5%	L00–L99	680–709
Diseases of the musculoskeletal system and connective tissue	446	54	12.1%	M00–M99	710–739
Diseases of the genitourinary system	1205	69	5.7%	N00–N99	580–629
Perinatal diseases	1	0	0.0%	P00–P96	760–779
Congenital disorders	19	2	10.5%	Q00–Q99	740–759
Symptoms, signs, abnormal findings, ill-defined causes	1764	89	5.0%	R00–R99	780–799
External causes of injury and poisoning	1232	95	7.7%	V01–Y89	E800–E999
Unknown cause of death**	181	16	8.8%		
Total	59760	3736	6.3%		

^*^Not shown “Complications of pregnancy, child birth and early maternity”

^**^Officially not part of the BELDO list

The highest autopsy rate was observed when the death certificate showed a COD related to the digestive system (16.9%), followed by infectious and parasitic diseases (14.3%). Table 3 shows the most common individual COD within the groups with the highest and the lowest autopsy rates. The diseases of the digestive system consisted of many different large subgroups with an autopsy rate of > 10%, while infectious and parasitic diseases consisted of a large subgroup ‘other sepsis’ (49.0%) with an autopsy rate of 18.8%.

**Table 3:** Most common individual COD in the groups with the highest and lowest autopsy rate

Individual COD	*N* deaths	% deaths of total in group	Autopsy rate	ICD-10
Highest autopsy rate				
Diseases of the digestive system (16.9%)				
Other diseases of the digestive system	388	18.6%	11.3%	K92
Paralytic diseases and intestinal obstruction without hernia	237	11.4%	17.3%	K56
Vascular disorders of the intestine	194	9.3%	22.2%	K55
Diverticular disease of intestine	158	7.6%	15.8%	K57
Other diseases of intestine	144	6.9%	22.2%	K63
Fibrosis and cirrhosis of liver	138	6.6%	10.1%	K74
Infectious and parasitic diseases (14.3%)				
Other sepsis	352	49.0%	18.8%	A41
Other and unspecified infectious diseases	108	15.0%	1.9%	B99
Lowest autopsy rate				
Mental and behavioral disorders (0.8%)				
Unspecified dementia	1957	83.6%	0.6%	F03
Vascular dementia	313	13.3%	0.6%	F01
Diseases of the nervous system and the sense organs (4.0%)				
Parkinson disease	661	38.7%	2.7%	G20
Alzheimer disease	414	24.2%	0.5%	G30
Spinal muscular atrophy and related syndromes	190	11.1%	10.0%	G12
Multiple sclerosis	61	3.6%	1.6%	G35

Next, the least autopsies were performed in mental and behavioral diseases (0.8%) and diseases of the nervous system and the sense organs (4.0%). By far, the largest subgroup within the mental and behavioral diseases concerns the “unspecified dementia” (83.6%) followed by “vascular dementia” (13.3%), with both having an autopsy rate of 0.6%. Diseases of the nervous system and the sense organs mostly concerned “Parkinson’s disease” (38.7%) and “Alzheimer’s disease” (24.2%) with autopsy rates of 2.7% and 0.5%, respectively.

As shown in Table 4, the autopsy rate increased with an increasing number of contributing COD per deceased person. The autopsy rate for a deceased person with one declared COD was 5.2%. In contrast, the autopsy rate for a COD with three contributing causes of death was 8.0%. The independent sample *t*-test to compare the number of CODs in deceased with (1.72) and without (1.88) autopsies showed *p* < 0.001.

**Table 4:** Autopsy rate according to number of recorded causes of death

Number of recorded COD	*N* deaths	*N* autopsies	Autopsy rate
Only underlying COD	30064	1556	5.2%
One contributing COD	18590	1269	6.8%
Two contributing COD	7769	641	8.3%
Three contributing COD	3156	254	8.0%
COD unknown	181	16	8.8%
Total	59760	3720	6.2%

The autopsy rate after a clinical diagnosis of cancer is shown in Table 5. The autopsy rate in deceased without a clinical diagnosis of cancer was 4.4%. The overall autopsy rate for deceased with a previous clinical diagnosis of cancer was 6.2%. A higher or lower O/E ratio, respectively, signifies a higher or lower autopsy rate than expected, based on the autopsy rates per age category in deceased without a diagnosis of cancer. Incidental findings during the autopsy could not be excluded in the “0–1 days” group, which shows an autopsy rate of 78.0%. These included cancer of the lung, prostate, kidney, pancreas, large intestines, and lymphatic malignancies such as acute lymphocytic leukemia, non-Hodgkin, and Hodgkin lymphoma. When we excluded the first 0–1 days, the autopsy rate was 5.2%. There was a higher autopsy rate than expected in the first month (2–31 days) after a diagnosis of cancer, a slightly lower autopsy rate than expected in the months and years thereafter, and a stabilization after approximately 15 years. Most deaths occurred within 1 year after a diagnosis of cancer (*n* = 9363), mostly within the “32–183 days” category (48%).

**Table 5:** Observed/expected ratio of autopsies according to duration after cancer diagnosis*

			*N* deaths	*N* autopsies	Autopsy rate
No clinical cancer diagnosis			55,493	2443	4.4%
Clinical cancer diagnosis overall			24,971**	1550	6.2%
Cinical cancer diagnosis excluding 0–1 days			24,626	1281	5.2%
		Autopsies			
Time between cancer diagnosis and death	*N* deaths	*N* observed	*N* expected	O/E ratio	95% CI
0–1 days	345	269	22,7	11,84	10.42–13.25
2–31 days	1853	287	123,1	2,33	2.06–2.60
32–183 days	4491	275	313,3	0,88	0.70–0.98
184–365 days	2674	132	189,4	0,77	0.58–0.82
1– < 2 years	3068	139	206,7	0,67	0.56–0.78
2– < 3 years	1948	81	120,2	0,67	0.53–0.82
3– < 4 years	1511	76	84,1	0,90	0.70–1.11
4– < 5 years	1218	52	64,3	0,81	0.59–1.03
5– < 10 years	3955	138	178,0	0,78	0.65–0.90
10– < 15 years	2116	56	73,6	0,76	0.56–0.96
15– < 20 years	1105	30	30,8	0,97	0.63–1.32
20 + years	687	15	16,8	0,89	0.44–1.34
Total	24,971	1550	1423,0	1,09	1.03–1.14

^*^Not using NLCS data, so inclusion of autopsies from 1991–2014

^**^Excluding one case with missing information on date of diagnosis

## Discussion

Our study investigates the relation between the clinical cause of death, a history of cancer, and the autopsy rate by linking the NLCS to nationwide databases: the cancer registry, the Dutch Nationwide Pathology Databank (PALGA), the population registry, and the cause of death registry. The cause of death registry uses death certificates that are completed by physicians, usually within a few hours after a patient dies and before an autopsy is performed. To our knowledge, this linkage between clinical COD and the autopsy rate has not been investigated before. As in most European countries, permission for a clinical autopsy needs to be consented to by the relatives. In the Netherlands, this is done orally. This presumably results in more reluctance compared to countries in which no consent is needed, or autopsies are obligatory, especially in the older age categories. Consequently, in the Netherlands, the autopsy rate is among the lowest in Europe [6]. In a national study encompassing all clinical autopsies in the Netherlands, the autopsy rate in the age category 60–79 declined from just below 10% to approximately 3.9% from 1991 to 2015. In addition, the autopsy rate varied between different age categories, i.e., an average autopsy rate of 8.06% in the age category 60–64 years and 1.12% in 90–94 years. Lastly, a difference in autopsy rate according to sex could be explained by the age difference at the time of death. Our study shows a similar decreasing autopsy rate with older age, as shown in Table 1. More than half of all participants died from 1991 to 2009, mostly between the ages of 65 and 84 years. Contrarily, in all age categories, the autopsy rate was higher in males.

The results show a varying autopsy rate with different CODs, as shown in Table 2. Some considerations can be made when looking at the ICD codes that constitute the BELDO list, as shown in Tables 2 and 3. The autopsy rate in different CODs is derived from the NLCS, which is a population aged 55–70 at baseline in 1986, with a follow-up of 23 years. Although most deaths in the Netherlands occur in these age groups, these findings cannot be extrapolated to all patients as the COD distribution varies with age.

The most important consideration for relatives to give permission for an autopsy is the wish to learn about the cause of death [18]. The highest autopsy rate was observed in COD related to the digestive system (16.9%), followed by infectious and parasitic diseases (14.3%). Infectious and parasitic diseases can develop quite fast and are unpredictable, but as a CODs, they are rather rare in the Netherlands. This might explain the relatively high autopsy rate. One would expect this number to have increased even more in the face of the COVID-19 pandemic, but this is outside the time frame of this study. One could argue diseases of the digestive system may cause vague symptoms, which might lead to an unexpected and/or unexplained demise, therefore increasing the “need to know” of relatives and physicians.

Next, the autopsy rate was lowest in mental and behavioral diseases (0.8%) and diseases of the nervous system and the sense organs (4.0%). Of all the deaths in the “mental and behavioral diseases” group, 83.6% consisted of “unspecified dementia,” which was rated number 7 in the list of most common causes of death in 2000 in the Netherlands [32]. As patients with dementia are more prone to demise at home or in a nursing home, this might lead to a difference in autopsy rate, as shown by Lindstrom [3]. In addition, due to the long process of the disease, next of kin and physicians might be less inclined to refer to an autopsy [18]. A similar explanation is applicable to Alzheimer’s and Parkinson’s diseases in the group “diseases of the nervous system and sense organs.” An important reason for not requesting or permitting an autopsy is the assumption that the cause of death is already known [18]. This might be a feasible explanation for the low autopsy rate in deaths due to dementia, Parkinson’s disease, and Alzheimer’s disease. For the sake of completeness, there was one death due to an unspecified perinatal disease of the digestive tract, without an autopsy, which could either be a coding error or a very late death because of a congenital abnormality.

Our results show that the autopsy rate was positively correlated with the number of contributing causes of death (Table 4). This suggests physicians are more likely to request an autopsy when confronted with complex cases. However, due to technical and/or legal limits, death certificates are mostly completed before the conclusions of a postmortem become available. Despite improvements in clinical healthcare and technical advances in the last decades, a significant amount of major and minor discrepancies between clinical diagnoses and autopsy findings still remain [7, 20-22, 24, 33]. Major discrepancies are findings associated with the COD, where prior knowledge ante mortem might have changed patient management and survival in some cases. A study in the Netherlands showed major discrepancies in 16% of the autopsies in 2012/2013 [19]. Thus, autopsies continue to provide invaluable information for medical education and quality assurance [1]. Autopsies in particular have also been used as a measure for the accuracy of death certificates in general populations [34, 35] and in selected groups of diseases [36]. Therefore, some authors suggested that death certificates should be completed or amended utilizing data gained during autopsy [37]. Death certificates are the main source for mortality statistics and, as an indicator, contribute greatly to detecting trends in (inter)national healthcare [38]. As physicians are required to complete the death certificate, they play an important role in mortality statistics, and therefore indirectly, in the distribution of resources in healthcare and research.

In our study, the autopsy rate was affected by a diagnosis of cancer, most dramatically in deaths within 31 days after a diagnosis of cancer (Table 5). This increase in the first month could be explained by a sudden unset and/or rapid increase of the cancer, which may lead to unanswered questions for physicians and relatives. In the months and years thereafter, the cancer and possible cause of death would be known, which could lead to a decreased interest in autopsies, up to the stabilization after some 15 years.

The effect of completing death certificates without autopsy results, in regard to the incidence of cancer, is inconsistent. A study from 1997 conducted in Sweden suggested that the incomplete postmortem information due to the decline of autopsies was associated with a difference in the registered incidence of cancer [3]. However, in another study published in 2015 in Switzerland [39], the total registered incidence of cancers was not affected by the lower autopsy rate, perhaps due to advances in modern diagnostic tools as suggested by the authors. Another explanation for the contrasting results of both studies might be methodological and structural differences in the organization of the cancer registries within the two healthcare systems. In our study, a diagnosis on days 0–1 most likely means the cancer was detected as an incidental finding, i.e., during the autopsy, and was thereafter recorded in the cancer registry. Alternatively, the cancer was identified during a medical procedure such as surgery, after which the patient died, and the diagnosis was confirmed during an autopsy. In other words, the cancer was presumably undiagnosed before passing away in most of these cases. This probably explains the significantly higher autopsy rate of 78.0% on days 0–1. As there are only 269 autopsies with a diagnosis of cancer on day 0–1 in a study group with almost 25,000 patients with cancer, a possible effect of a decreasing autopsy rate on the cancer incidence is limited. Blokker et al. [40] speculated that the lower autopsy rate in older patients might be correlated to an increased number of deaths due to cancer. This differs from the findings presented here, as our average autopsy rate in cancer patients was slightly higher than in patients without cancer, even after excluding the “0–1 days” group. Our results suggest that the lower autopsy rate in older patients is more likely due to death in cases of dementia, as discussed above.

In our study, the linkage with excerpts from the PALGA-database was used to investigate whether an autopsy was performed. Not being able to see the full autopsy report in PALGA is therefore a limitation. Access to this data, perhaps in comparison with the cause of death registry by the CBS, may provide additional interesting insights.

The relevance of autopsies has been described in numerous publications over the years. Although major discrepancies between autopsies and clinical diagnoses remain [19–25], a steady worldwide decline of autopsies has been observed [3–6]. Therefore, it is the opinion of the authors that medical healthcare in general, as well as individuals, would benefit from an increase in postmortem investigations, among which autopsies. This increase can only be effectuated in close collaboration with clinicians and should be a solution for a problem, not the mere goal.

## Conclusion

This study shows the relation between the clinical cause of death, a history of cancer, and the medical autopsy rate in a large national cohort. There were major variations in the autopsy rate in relation to the clinically reported cause of death. The autopsy rate was positively correlated with the number of contributing causes of death, suggesting a higher interest in autopsies in complex medical cases. Lastly, the presence of cancer only showed an increased autopsy rate in the first year after diagnosis. The insight this study provides may help clinicians and pathologists to understand the decreasing autopsy rate and intervene in the further downfall of the medical autopsy.
